# Twelve Tips for Better Communication with Patients During History-Taking

**DOI:** 10.1100/tsw.2007.73

**Published:** 2007-04-30

**Authors:** Aminur Rahman, Saria Tasnim

**Affiliations:** ^1^ Center for Injury Prevention and Research, Bangladesh (CIPRB), House 226, Lake Road 15, New DOHS, Mohakhali, Dhaka 1206, Bangladesh; ^2^ Department of Obstetrics and Gynaecology, Institute of Child and Mother Health (ICMH), Matuail, Dhaka 1362, Bangladesh

**Keywords:** history taking, communication, patients

## Abstract

Eliciting proper history from a patient is of paramount importance to establish an accurate diagnosis and management in medical practice. Good communication skill is a prerequisite for an effective physician patient relationship. A systematic search of medical literature has been made to formulate a guideline for better communication during history taking. The guideline emphasizes on both physical environment and emotional encounter and the key points are expressed as tips on relevant issues.

## INTRODUCTION

The history obtained from a patient is usually of paramount importance in medicine and to elicit a proper history, adequate communication skills are necessary[1,2]. The accuracy of diagnoses and the establishment of therapeutic physician-patient relationships depend on effective communication skills within the medical interview[3]. However, this is the very aspect of physicians' professional skills that is still the most common cause of complaint from patients or their relatives, which can be seen as an apparent weak point in physicians' professional competence[4].

## METHODS

A systemic search for medical literature was performed using computerized databases including Medline to find out the ways for better communication between physician and patient. The search parameters were revised several times by adding or deleting key words to assure a complete review. In addition, the citations of articles identified through computer searches were examined. Moreover, experts were also contacted to identify other work that may have missed. The results of review were formulated into twelve tips for better communication.

## BEGINNING OF A HISTORY TAKING SESSION

The patient's impression of the physician is generally formed within minutes of commencing the interaction and this is the stage where rapport is established between physician and patient, which helps the patient to open up[5,6].

*Tip 1: Make sure that your beginning is smooth. All you need to do is – greet the patient, check the identity of the patient, introduce yourself, offer some personal but interesting and relevant chit chat, and finally do not forget to explain the purpose of the interview.*

## SEATING AND BODY POSTURE

Communication can be helped or hindered by the physical setting where the interview is conducted[4]. Normally, patient and physician should be comfortably seated at the same level so that the eyes of the physician are on level with the patient[4,7]. In hospitals this can be easily achieved by sitting down on a chair beside the patient's bed[8]. Sitting down sends some important signals to the patient-that the physician is there to listen, that he/she is under the control of the patient and that he/she would like to engage in non-patronizing communication. Furthermore, when the physician sits down, the patient perceives the length of time spent at the bedside as longer than if the physician remained standing.

To avoid direct face to face confrontation the seating arrangement should be set at an angle to each other, rather than directly opposite[4,9]. An ideal distance between the patient and the physician would be around one meter, reflecting something between intimate and casual or personal distance[10], however, this distance seems to vary from culture to culture[7]. The arrangement should be close enough to convey confidentiality, but not so close as to intrude upon each other's personal space[4].

When seated, a forward leaning posture by the physician has been found to be associated with higher patient satisfaction[11]. An open posture with hands and legs uncrossed tends to be interpreted as a signal of warmth, acceptance and willingness to participate[12] and is the most facilitative posture[13].

*Tip 2: Plan a facilitative seating arrangement and maintain forward leaning open posture.*

## EYE CONTACT

Eye contact is very important since it is usually a pre-requisite for any conversational interaction. Frequent but not constant eye contract facilitates communication[8]. It conveys a willingness to participate with another person. Constant eye contract is experienced as aggressive or erotic[13]. No eye contact may convey embarrassment, disinterest or deception. When dealing with patients, it is important to display appropriate levels of eye contact, since too much or too little can be disconcerting[8].

*Tip 3: Display appropriate level of eye contact; do not over use or under use.*

## LISTENING

One of the common misdemeanors is that the physician does not let the patient talk. Detailed studies of physician interviews with patients revealed that patients usually attended physicians with between 1.2-3.9 major complaints. However, the time that the patient was allowed to talk before being interrupted by the physician was eighteen seconds and they only ever finish their opening statements in 23% of cases[14].

Effective listening does improve patient satisfaction[15] particularly if they are allowed to tell their own story in their own words and express their feelings[16,17]. During interpersonal encounters, the practitioner must make the effort not only to listen to others, but also to clearly demonstrate, both verbally and nonverbally, that he is indeed doing so[18]. This type of active listening is also required to ensure that maximum verbal and nonverbal information is received from patients[8].

Physicians often fail to keep to relevant matters by using proper reinforcement, reflection and interrupting when the patient is "off-the –track". However, the patient should only be interrupted when absolutely necessary[7].

*Tip 4: Interrupt less frequently, listen carefully and keep the patient to relevant matters.*

## USE OF FACILITATION

Providing proper facilitation on the part of the physician is essential for effective communication to occur. Facilitation can be provided in both non-verbal and verbal ways. Non-verbal ways include - smiling, nodding, and showing an unhurried manner, using appropriate body language and also appropriate touching of the patient. The verbal types of facilitation are- reflection i.e. the physician encourages the patient to continue speaking by repeating, and so reflecting back to him, a phrase, idea or significant word from what the patient has just said by which the patient is prompted to express himself further[4]. The reinforcement is used in interviewing to encourage the involvement of the other person, to demonstrate interest, to develop and maintain relationships, to provide reassurance to convey warmth and friendliness and to help to control the topic of conversation. There are wide ranges of verbal and nonverbal behaviors, which are employed in order to reinforce others[8].

*Tip 5: Use of frequent facilitation to encourage the patient to talk and to stay involved in the conversation.*

## SILENCE

Silence can occur during history taking, when the patient runs out of words or is unsure about expressing his/her feelings. In this situation, the physician must resist the temptation to fill the silence instantly with a new question on a new topic. A silence usually means that the patient is thinking or feeling something important, not that he or she has stopped thinking. So the patient should be given a little time with an expression of unhurried interest and concern[4,7].

*Tip 6: "Silence is golden"- so tolerate short silences. The patient may be doing important 'work'.*

## PSYCHOSOCIAL AND PERSONAL ISSUES

Psychological and social factors have obvious impacts on patients' illness states[19]. However, many physicians begin to focus on patient symptoms and restrict themselves to functional enquiries. They are reluctant to ask about relevant psychological and social aspects of their histories. As a result they receive very little information about psychological and social factors, which are so important in-patient care[1,19,20].

Eliciting sensitive and personal information is central to the performance of effective and complete care. Yet it is taxing to both physician and patient[21]. However, the patient and physician must both overcome prior conditioning that personal and sensitive information is not welcomed or considered relevant by physicians. The fact that painful personal material taxes the sick person is often true, but it is also often helpful or necessary. Physicians sometimes rationale that patients should not be subjected to invasive inquiry in order to avoid discomfort. Yet patients welcome such discussion more often than not. To elicit sensitive and personal information, the most effective way to begin demonstrating to the patient that personal feelings and experience are of importance in the process of care is to take the first opportunity to inquiry about them. After doing this several times, the patient will realize that the physician wants to know and understand more than a narrow account of disease[13].

*Tip 7: Attempt to include relevant psychosocial issues in the discussion and show your willingness to discuss emotional and highly personal issues raised by the patient.*

## EMPATHY AND WARMTH

Empathic responses do not signify that the physician would probably, if placed in similar circumstance, have similar feelings. But the physician is indicating that he shares them to the extent that he recognizes and understands them, and hinting that he would probably, if placed in similar circumstance have similar feelings[4]. However, physicians often exhibit a low level of empathy, when interviewing patients[22]. Sometimes this leads to a complete breakdown in communication, since patients become so preoccupied with their own unexpressed worries that they stop paying attention to the physician[23].

Patient satisfaction and compliance with treatment are positively related to perceptions of practitioners as being warm and friendly[24,25]. Thus it is important for practitioners to demonstrate a warm, friendly approach when dealing with patients[8]. Physicians or medical students express warmth, when they accept the patient as a person[26].

*Tip 8: Show your empathy and be warm and friendly by considering the patient as person, not just a clinical problem.*

## VERBAL AND NON-VERBAL LEADS

Patients frequently open the discussion with a presenting problem and will often reveal their real problems, when encouraged to do so. It is therefore important to be aware of verbal and non-verbal signals, which patients may emit to indicate a desire for a deeper level of discussion[27]. One criterion of skilled communication is the ability to recognize and act on non-verbal changes in the patient. In addition to this there are skills associated with the transmission of appropriate non-verbal signals to the patient. These can indicate interest, concern and empathy and be of great value in facilitating communication[28].

*Tip 9: Pick up leads from what the patient says or does; remember to follow the patient sometimes.*

## QUESTION STYLE

To elicit a proper history an appropriate use of questions is necessary. “Funneling” can be used, which refers to the use of questions to guide the conversation from the general to the specific i.e. the questioner uses open questioning initially and proceeds to probing questions and then closed questions[27].

*Tip 10: Start with open ended questions and proceeds to probe questions and then closed questions.*

## USE OF JARGON

Physicians are constantly tempted to use medical jargon, when they speak to patients[29]. Most often for the patient, jargon is an unintelligible language[7]. Studies showed that jargon confused and alienated the patients, often leading to misunderstanding[30] and misinterpretation[31].

*Tip 11: Avoid using jargon; use simple everyday language.*

## CONCLUSION OF THE INTERVIEW

In relation to physician-patient communication it is expected that 'bringing the consultation to an end should be a progressive, step by step process through which physician and patient will co-operate and co-ordinate their actions[32]. However, in the actual setting it is commonly observed that ending the interview is rushed over in order to get on to the next patient or activity[13,33].

*Tip 12: Do not show hurried mannerisms; provide a summary of the interview; ask the patient if any thing else is troubling him/her; invite questions from the patient; give a clear indication of the closure of the interview and finally reinforce the patient by providing appropriate remarks at the end of the interactions.*

## CONCLUSIONS

Proper communication with patients is an important skill for medical practice, yet less often addressed in the medical curriculum. History taking is the first step to patient management and twelve tips have been suggested to facilitate this encounter.
